# Seventh Annual Report of the Royal Commission

**Published:** 1884-01

**Authors:** 


Seventh Annual Report of the Royal Commission for veterinary matters, upon the extension of contagio-infectious animal diseases in Prussia, for the year ending March 31, 1883.

*Anthrax.*

Anthrax made its appearance on 643 farms; from it perished 39 horses; 907 cattle; 884 sheep; and 36 hogs.

The greatest number of cases of anthrax appeared in localities where the disease was known to be stationary, or to appear at intervals. The country flooded by the overflows of the Weichsel and Oder, in the spring was especially prolific in cases of anthrax in the second quarter of this year. Many cases of the disease were directly traceable to the animals being fed on hay gathered from known anthrax-infected localities. Such food was shown to have caused the disease in cattle, when it had been sold to owners at a distance from the place where it was gathered.

*Rabies.*

Rabies appeared in 649 places among 431 dogs; 7 horses; 87 cattle; 11 sheep; 17 hogs: 239 ownerless dogs were killed on suspicion of rabies, and 939 for other lawful reasons.

The disease was also developed in 7 cats during the year.

*Glanders and Farcy.*

This disease was developed in 611 different places during the year: the total number of horses in these stables was 4734, of which 1547 were diseased: of these 80 died; 1359 were killed by the authorities, and 129 by the owners.

The government paid to the owners 343056 marks ($85,764) for horses killed by the veterinary police. The average per horse being 2534 marks, or $63.50.

*Foot and Mouth Disease.*

This disease appeard among 28,455 cattle; 6,659 sheep, and 1,801 hogs.

The markets proved a general centre from which this disease became dispersed.

*Pleuro-Pneumonia Contagiosa.*

appeared in 214 places among 8875 head of cattle, of which 1,953 were diseased; of these 48 died, 1,757 were killed by the authorities and 274 by the owners.

The outbreaks at Berkenbrügge and Naulin are of especial interest to those interested in the veterinary police. In both places the disease again broke out after the greater part of the disease or suspected animals had been killed, and when no case of suspicion had appeared in the herds for a period of six months from the time the last case was developed which must lead to the opinion that some of the animals still retained the power to infect others though no symptoms of the disease had been observed in them during the six months quarantine; that is, from the time they were declared to have became free from disease.

When inoculation has been performed before the animals have been exposed to infection, it seems to act as a secure prophylaxis.

The costs for slaughtered animals were 512.186 marks.

Vesicular genital eruption in horses and cattle, appear in 141 horses, and 1,003 cattle.

The "Scab" attacked, 69,666 sheep.

* Two children (twins) were taken ill with a vesicular eruption of the mouth, etc., from the consumption of the milk of a goat which had 'foot and mouth disease.' The period of incubation was from three to five days in one, and six to seven in the other child.

The chief symptoms were, loss of appetite, vomiting, dysphagia, fever, vesicles on the oral mucosa, lips, and in the nose, which coalesced and formed ulcers in many places. One child died, the other recovered. B.

* Demme Zeitshift f. Theirmedcin, vol. 9, part 4.

